# Editorial Comment: Image-guided study of swine anatomy as a tool for urologic surgery research and training

**DOI:** 10.1590/S1677-5538.IBJU.2021.06.03

**Published:** 2021-10-01

**Authors:** Luciano A. Favorito

**Affiliations:** 1 Universidade do Estado do Rio de Janeiro Unidade de Pesquisa Urogenital Rio de JaneiroRJ Brasil Unidade de Pesquisa Urogenital - Universidade do Estado do Rio de Janeiro - Uerj, Rio de Janeiro, RJ, Brasil.

Jacob Hindrik Antunes Smit 1, Eduardo Piotto Leonardi 2, Rosa Helena de Figueiredo Chaves 3, Ismari Perini Furlaneto 3, Cezar Massoud Salame da Silva 4, et al.

Acta Cir Bras. 2021 Jan 20;35(12):e351208

DOI: 10.1590/ACB351208 | ACCESS: 10.1590/ACB351208

## COMMENT

In this interesting paper the authors shows the importance of the animal models for urological training. Basic research is very important to to provide the basis for training young urologists using human or animal models (1, 2). The use of pigs and dogs to show the anatomical aspects of urological structures is very well established in the literature (3, 4). In this paper the authors describe the anatomy of the swine urinary system using computed tomography and to discuss the role of this animal as an experimental model for urological procedures and concluded that the data obtained show similarities with human anatomy, suggesting the viability of the swine model for planning preclinical trials, basic research, refinement in experimental surgery and surgical training for urological procedures.
